# The International Medical Congress of 1900

**Published:** 1899-12

**Authors:** 


### THE INTERNATIONAL MEDICAL CONGRESS OF 1900.

PREPARATIONS on a large scale are being made for the International Medical Congress, to be held at Paris, during the Universal Exposition of 1900. A large separate building is being erected on the exposition grounds, in which the different congresses are to be held—on the same plan as adopted at Chicago in 1893. One of the most important sections of the Medical Congress will be that on medico-legal science, under the able presidency of Prof. P. Brouardel, Doyen of the University of Paris, and the leading authority on legal medicine in Europe. He has arranged a series of reports for discussion as follows :

1. Pulmonary Docimacy. Reports in French (French "Rapporteur"). Messrs. Descoust and Bordas.

2. Actions (the effects) of the new smokeless powder on garments and the skin. Reports of Messrs. Thoinot and Vieille.

3. Ocular putrefaction as a means to determine the date of death. Report of M. Descoust.

4. The criminal combustion of corpses. Report of M. Ogier.

5. On accidental death by electricity. Reports of Messrs. d'Arsonval and Bordas.

6. Valvular lesions consequent upon (resulting from) the traumatism of the walls (coats) of the thorax. Reports of Messrs. Castiaux and Langier.

7. Expert examination rendered necessary by the accidents which might result from the frequent use of foods or drinks which have been conserved by chemical agents (borax, salycilic acid, formol, etc., etc.). Reports of Professors Brouardel and Pouchet.

8. On crimes (offences) resulting from the practice of magnetism by persons who have no diplomas. Reports of Messrs. Gilles de la Tourette and Rocher.

Besides these reports papers will be read on other important aspects of legal medicine and a valuable meeting seems to be insured. An auxiliary committee has been appointed in this country with Mr.

Clark Bell, the well-known editor of the New York Medico-Legal Journal, as chairman. This section, so ably officered, so well organised, and having so many important subjects under discussion ought to be one of the best attended of all the various meetings scheduled for the exposition season.

---

THE Board of Aldermen passed the Christian Science resolution "after election," as the JOURNAL said they would. Now it is going through the Board of Councilmen, where it has had a hearing.

---

THE *New York Herald* sent a supply of yellow fever serum to Havana recently. The results of its use are given in a cable to the *Herald* from one of its correspondents, who, writing under the date of November 9th from Havana, says :

The Sanarelli serum sent here by the *New York Herald* was given a practical and highly satisfactory test last week. The two patients to whom it was administered have fully recovered from yellow fever, and they have been placed in the convalescent ward. The sanitary measures taken by the American government and the liberal use of the electrical disinfectant has kept down a yellow fever epidemic in the city proper, but along the water front there have been spasmodic cases, and the Machina wharf seems to have been a great breeding place for the germ.

It was there that last May the first fatal cases were developed in the Marine Corps, and two engineers and a deck hand of Admiral Cromwell's launch have since died there.

Dr. Raphael O. Marcour, of the United States Marine service, is the doctor in charge at the Machina. He is one of the youngest doctors in the service in Cuba, and has given yellow fever a special study. The several cases which come under his charge at the Machina were of a most virulent type, and nothing in the way of old remedies seemed to stop the progress of the disease.

The Sanarelli serum, which arrived in September, had been carefully laid aside by the staff of military doctors, who were tempted to look upon the use of serum as an experiment wholly outside of a military man's line. Last week, however, Dr. Marcour took the initiative.

Lawrence Fitzhugh, an assistant on the Admiral's launch, had been sick four days with a pronounced case of yellow fever. Black vomit had set in, and there was no hope by the ordinary treatment to save the man's life. Dr. Marcour applied to Maj. Gorgis for some of the *Herald's* Sanarelli serum.

That evening he injected some into the abdomen of Fitzhugh. In three hours there was a change for the better. The next day the black vomit stopped, and in three days all traces of yellow fever had left the patient. He was, however, in a very weak condition on account of his severe illness before the serum was administered, and it took careful nursing to bring him around.

While Fitzhugh was recovering, another man, the steward of Dr. Marcour, John H. Sublette, was taken ill, and he had severe symptoms of the fever that had proved so fatal to the occupants of the Machina. Dr. Marcour, elated at the pronounced success in the case of Fitzhugh, immediately gave Sublette an injection of the serum, and from the moment it began to work on his system improvement in his condition was noticeable. The two men are fast recovering, and the doctor says that they will be at work again in a few days.

There is no doubt in the mind of Dr. Marcour that the Sanarelli serum is an antitoxin for yellow fever, and he has strongly urged its use in the yellow fever hospital here. He furthermore firmly believes that the serum is a preventive as well as a cure for the fever, and he is making arrangements to vaccinate one of the men on the Admiral's launch who has consented to submit to the operation.

It is shown by the experiments of Dr. Eugene Wasden, who was conducting the work in the yellow fever government experimental station here last spring, that the Sanarelli serum, when injected into rabbits and guinea-pigs used as subjects, caused a mild case of yellow fever, and a man treated similarly would be to all intents and purposes immune.

---

THE hearing before the councilmen lately held relative to the ordinance opposed by the faith healers was the most interesting as well as the clearest of all the many seances on that subject. Mr. Tracy C. Becker, of counsel for the Medical Society of the County of Erie, was particularly happy in the part he played.

He pointed out in unmistakable English that the language of the present ordinance is of very uncertain meaning, and declared: "As a lawyer, I doubt whether any person could be convicted under that section under any circumstances."

This ought to afford sufficient ground for the councilmen and aldermen alike to act promptly in framing a new ordinance that should compel all citizens who have knowledge on the subject, no matter how acquired, to report to the board of health all contagious diseases. No doubt it will have that effect.

As to the resolution proposed by the aldermen to soothe the perturbed spirits of the healers, it was natural for "Judge" Cunneen to contend for its adoption, for it is all he will have to show for the fee, presumed to be an ample one, that he receives for his services.

---

THE United States Marine Hospital Service has lately sent out a report giving the vital statistics of American cities during the year

1898. From this it appears that the death-rate in the State of New York was 18.46. If, however, the City of New York be left out of the account, the death-rate for the state, as a whole, would be somewhat lower.

Pursuing the examination of this report still further, and coming down to particular cities, we note that Buffalo is recorded as one of the most healthful of them all.

The Buffalo death-rate last year was 12.24. This reckoning is obtained on the basis of an estimated population of 370,000, the number of deaths from all causes during the year being 4,533. No city in the United States of equal size shows a death-rate so low. It is noticeable that nearly all the lake cities were comparatively healthful. Cleveland, with a population also estimated at 370,000, had a death-rate of 13.62.

Buffalo has reason to be proud of her record of healthfulness as officially indicated in this report, a condition directly referable to its efficient health department.

---

The question of substitution is one of great interest and importance to physicians. A well-known house has recently brought to trial a druggist charged with the offense, and the following recent action of the court cannot fail to be of interest:

> A decision of considerable importance (*Chicago Times-Herald*, October 13, 1899,) was made by Judge Kohlsaat in the United States Circuit Court yesterday. In a bill for an injunction Fairchild Brothers & Foster, of New York, had charged Edward Otto, a Chicago druggist, with substituting a spurious and inferior preparation for "Fairchild's essence of pepsine" in several cases where the latter was expressly called for in physicians' prescriptions. The case was hotly contested and hundreds of pages of depositions were taken in New York and Chicago. Judge Kohlsaat's decree sustains the charges made, perpetually enjoins Otto from ever repeating the offense and taxes him with the costs, amounting to about $500. This is said to be the first contested case in the United States in which the principle of protection to trade-marks and trade names was extended so as to apply to what is technically known in the drug business as "substitution." Judge Kohlsaat's decision will probably protect manufacturing chemists, physicians and the general public, all of whom have in the past suffered from these fraudulent practices of a certain class of druggists.

The medical profession as well as druggists and pharmacists owe to Messrs. Fairchild Brothers & Foster a debt of gratitude for pushing this matter to a conclusive test.

IT IS reported that the Boers returned the British wounded recently delivered at Ladysmith without demanding equivalent in exchange. This, if true, is a commendable act of humanity; but it is more than probable that the act relieved the Boers quite as much as it pleased the English.

---

THE Major-Generals that edit the newspapers are continuing their hostile criticisms with a venomous sarcasm that sometimes borders upon positive disloyalty. An amusing instance of "smartness" was recently exhibited by a Buffalo paper that published an editorial entitled A Fruitless Victory, in which it criticised General Wheaton for withdrawing his troops after the San Jacinto affair. These are in part the words used :

It is said that the withdrawal was necessary because the roads were so bad that supplies could not be brought up. It is not easy to understand why supplies could not have been transported over roads upon which the soldiers themselves could march, especially when there were men and materials at hand to improve the roads.

Many a civil war soldier will smile at the stupidity of such a criticism. What novitiate in war does not know that soldiers can be moved forward under conditions that often preclude the transportation of supplies ? This same newspaper printed about the same time dispatches stating the amount of rain falling in November in Luzon was unprecedented.

Even soldiers have to eat once in a while and occasionally they have to be resupplied with ammunition.

The war portfolio and its subordinates, even the administration, can afford sometimes to be delivered from its friends.

---

THE report of Surgeon-General Sternberg on the care and health of United States troops shows that all the Atkinsonesque criticism of the blue-Monday shouters was cut on the bias. The Surgeon-General has good cause to be proud of the record made by his office and his corps during the war.

---

RECORDS in the Health Department show that there are an unusual number of cases of measles and scarlet fever prevalent in the city. The cases greatly outnumber those of the same period for last year. A comparison of the figures for October of last year and the first ten days of November, show that there were 77 cases of scarlet fever,

106 cases of diphtheria, and only one case of measles. For the same period this year there are 145 cases of scarlet fever, 77 cases of diphtheria, and 169 cases of measles.

Reports of measles have been made at the Health Department at the rate of 20 to 30 a day. Most of the cases are in the east side of the city, along Broadway, Seneca, Lovejoy, Adams, Emslie, William and Watson Streets. Thus far only four cases have been reported on the west side during October and to the middle of November of this year. These cases are on Lafayette, Auburn and Plymouth Avenues, and on Grant Street.

Every precaution is being taken by the health authorities to stamp out the disease, but the great danger lies in the neglect of parents to keep their children at home until they have fully recovered. Several instances have been discovered where people have neglected to report cases, but investigation showed it was the result of not knowing that a disease existed, rather than an attempt to violate or evade the law.

Four deaths due to measles complications have been reported.

---

SURGEON-GENERAL STERNBERG'S annual report gives a comprehensive view of the health of the troops, in the field and at home, the casualties and losses in battle and in hospital, the care extended to the sick and wounded, and other interesting information relating to the physical welfare of the army.

General Sternberg says it has been his endeavor that the sick and wounded should be supplied with every comfort and restorative, and the surgeons with every appliance that modern science would suggest in the treatment of disease and injury.

The total number of deaths in our armies, including regulars and volunteers, from May 1, 1898, to June 30, 1899, was 6,619, of whom 496 were killed in battle, 216 killed by accident, 202 died of gunshot wounds and wounds received in action, 2,774 from typhoid fever, 476 from malaria fever, 359 from pneumonia, 342 from diarrhea and dysentery and 185 from yellow fever.

He refers to the agitation over canned roast beef and refrigerated beef, and says at that time there were on file only two complaints as to the beef supply. The only criticism made by medical officers, he says, is that the ration for the tropics should have less fat and more starch and sugar.

